# Telehealth and cardiometabolic-based chronic disease: optimizing preventive care in forcibly displaced migrant populations

**DOI:** 10.1186/s41043-023-00418-x

**Published:** 2023-09-04

**Authors:** Ramfis Nieto-Martínez, Diana De Oliveira-Gomes, Juan P. Gonzalez-Rivas, Tala Al-Rousan, Jeffrey I. Mechanick, Goodarz Danaei, The Migrants Health Lown Scholar Program Team

**Affiliations:** 1 Precision Care Clinic Corp., Saint Cloud, FL USA; 2 Departments of Global Health and Population and Epidemiology, Harvard TH Chan School of Public Health, Boston, MA USA; 3 Foundation for Clinic, Public Health, and Epidemiology Research of Venezuela (FISPEVEN INC), Caracas, Venezuela; 4 Department of Internal Medicine, University of Texas Southwestern Medical Center, Dallas, TX USA; 5 International Clinical Research Centre (ICRC), St Anne’s University Hospital Brno (FNUSA), Brno, Czech Republic; 6 Herbert Wertheim School of Public Health and Human Longevity Science, University of California San Diego, La Jolla, CA USA; 7 The Marie-Josée and Henry R. Kravis Center for Clinical Cardiovascular Health at Mount Sinai Heart, Icahn School of Medicine at Mount Sinai, New York, NY USA

**Keywords:** Cardiometabolic, Chronic Disease, Hypertension, Migrants, Preventive medicine, Telehealth, Type 2 diabetes

## Abstract

The number of migrants, which includes forcibly displaced refugees, asylum seekers, and undocumented persons, is increasing worldwide. The global migrant population is heterogeneous in terms of medical conditions and vulnerability resulting from non-optimal metabolic risk factors in the country of origin (e.g., abnormal adiposity, dysglycemia, hypertension, and dyslipidemia), adverse travel conditions and the resulting stress, poverty, and anxiety, and varying effects of acculturation and access to healthcare services in the country of destination. Therefore, many of these migrants develop a high risk for cardiovascular disease and face the significant challenge of overcoming economic and health system barriers to accessing quality healthcare. In the host countries, healthcare professionals experience difficulties providing care to migrants, including cultural and language barriers, and limited institutional capacities, especially for those with non-legal status. Telehealth is an effective strategy to mitigate cardiometabolic risk factors primarily by promoting healthy lifestyle changes and pharmacotherapeutic adjustments. In this descriptive review, the role of telehealth in preventing the development and progression of cardiometabolic disease is explored with a specific focus on type 2 diabetes and hypertension in forcibly displaced migrants. Until now, there are few studies showing that culturally adapted telehealth services can decrease the burden of T2D and HTN. Despite study limitations, telehealth outcomes are comparable to those of traditional health care with the advantages of having better accessibility for difficult-to-reach populations such as forcibly displaced migrants and reducing healthcare associated costs. More prospective studies implementing telemedicine strategies to treat cardiometabolic disease burden in migrant populations are needed.

## The current global migrant crisis and the challenge of providing healthcare to migrants

International migrants include people who have chosen to migrate (e.g., economic reasons, study abroad, and family reunions) and those who are forcibly displaced due to conflict, persecution, or environmental disasters (e.g., refugees, asylum seekers, and undocumented persons) and are increasing in number in an unprecedented fashion [1], from 153 million in 1990, reaching nearly 272 million in 2019 [2]. Of these, the United Nations Refugee Agency estimates that global refugees surpassed 101.1 million in June 2022, with more than 2/3 of them coming from only six countries (Syria, Venezuela, Afghanistan, South Sudan, Myanmar, and Ukraine) and almost 40% of the forcibly displaced hosted in five countries (Turkey, Colombia, Uganda, Pakistan, Poland, and Germany) [3]. Of note, Syria (6.8 million) [3], Venezuela (6.13 million) [4], and Ukraine (5.8 million) [5] have the highest worldwide figures for people who leave the country. Migrants, especially the forcibly displaced populations, often face difficulties accessing quality healthcare in the country of destination, resulting in a higher risk and poorer control of noncommunicable diseases [6]. However, from an economic perspective, even when the incremental burden of healthcare for a forcibly displaced population is taken into account, restricting access to quality healthcare introduces more cost over time due to the incumbrance of more chronic diseases [7].

The migrant population around the world is heterogeneous in terms of vulnerability, medical needs, and access to quality health care. Among these, the forcibly displaced migrant population includes people at cardiometabolic and psychosocial risk that need a healthcare system infrastructure that can manage complex chronic disease scenarios. These scenarios include multimorbidity preventive care approaches [8]; continuity of care in humanitarian crisis settings; community-based management, engagement, and outreach; and health promotion [9, 10]. Vulnerability not only relates to non-communicable diseases, infectious diseases, and mental health issues developed within the country of origin, but also to adverse effects of travel conditions and acculturation challenges once in the country of destination. The aggregate result presents an enormous challenge for governments and the healthcare systems of the country of destination [11]. Low- and middle-income countries are particularly at risk, but even high-income countries can have limited capacity to provide culturally-sensitive healthcare to migrant populations [10]. Moreover, there is a conspicuous lack of reliable data to monitor the health of refugees and migrants, further impairing the ability of a country of destination to adapt and improve population health in this setting.

The World Health Organization [12] and the Boston Declaration [13] have emphasized the need for more epidemiological studies of chronic diseases and the development of standardized indicators in migrant populations, particularly as a result of humanitarian crises. Currently, a forcibly displaced migrant population tends to be more affected by chronic diseases, such as type 2 diabetes (T2D), hypertension (HTN), cardiovascular disease (CVD), and cancer, than infectious diseases [14]. In a recent meta-analysis, the pooled incidence of CVD in refugees from Syria and Libya to Europe was odds ratio (OR): 1.71; 95% confidence interval (95% CI): 1.03—2.83 times higher compared with non-refugee counterparts. Various factors can promote negative health outcomes: the interruption of medical treatment and preventive care during the migratory route and post-resettlement [15]; stressful situations, racism, and xenophobia [16]; hostile environment policies [17]; and living in refugee camps [18]. Additionally, forcibly displaced migrant populations have a higher risk of having trauma-related mental health problems [19]. The management of these conditions is challenged by several barriers, including limited access to quality healthcare and linguistic/cultural barriers experienced by patients and healthcare professionals (HCPs) [19–21].

In the USA, the forcibly displaced migrant population has a higher prevalence of chronic conditions than the general population, particularly T2D and HTN [22]. Specifically, the health of refugee populations in the USA beyond the first 8 months after arrival had higher odds of chronic diseases and T2D compared with non-refugee immigrant adults and US-born controls, respectively [22]. In a cross-sectional analysis of self-reported data, Kumar et al. [23] found that of 19,167 special immigrant visa holders from Iraq and Afghanistan in the USA, 56.5% had overweight or obesity, 19.4% were previous tobacco users, 2.4% had HTN, and 1.1% had T2D. However, these conditions may be underreported since full diagnostic workup is not conducted before travel to USA.

A meta-analysis of studies from Denmark and the USA [24] reported an increased risk of CVD in forcibly displaced migrant populations of both sexes compared to non-refugee migrants or native populations. In the US refugee clinic, it was found that 46.7% and 49.2% of this population had HTN or T2D, respectively, with more than one-quarter suffering from hyperlipidemia [24]. Moreover, the length of stay in the country of destination was associated with worse cardiovascular outcomes. Reported risk factors individually associated with an increased CVD risk in young refugees included socioeconomic status (e.g., neighborhood characteristics, income, and unemployment), as well as crime rate, welfare participation, and education. Nevertheless, one of the studies found that mortality in refugees younger than 40 years old was higher than in their country of origin, with CVD as the most common cause [24]. In this review, the aims are: [1] to describe the importance of culturally adapted interventions using as example the transcultural cardiometabolic-based chronic disease model [2], to describe the role of telehealth as a tool to improve the health of migrants, especially focused on cardiometabolic disease and lifestyle, and [3] to review the literature about telehealth interventions targeting HTN and T2D in migrants.

## The importance of culturally adapted interventions for migrant populations and the application of the transcultural cardiometabolic-based chronic disease model

The complex nature of interactions of biological and social determinants of health (SDOH) in vulnerable migrant populations requires a suitable pathophysiological model to guide optimal care. The transcultural cardiometabolic-based chronic disease (tCMBCD) model is such a model that contains three dimensions to facilitate early and sustainable preventive care [25–27]. The first dimension is a progression of chronic disease over time, consisting of four intuitive, distinct, evidence-based, and targetable stages: 1—“risk” (amenable to primordial prevention of risk development and progression); 2—“predisease” (amenable to primary prevention of disease); 3—“disease” (amenable to secondary prevention of complications); and 4—“complications” (amenable to tertiary prevention of suffering and mortality). Of note, stages may revert to an antecedent stage (e.g., prediabetes to euglycemia) or skip stages (e.g., insulin resistance to macrovascular complications, or prediabetes to microvascular/cardiovascular complications) [27]. The second dimension is based on a systems/networking effect of primary (genetics, environment, and behavior; together yielding a personalized lifestyle) and secondary/metabolic drivers (abnormal adiposity, dysglycemia, HTN, and dyslipidemia) [28]. The four secondary/metabolic drivers are interpreted individually according to the aforementioned stages as: adiposity-based chronic disease (ABCD) [29], dysglycemia-based chronic disease (DBCD) [30], hypertension-based chronic disease (HBCD), and lipid-based chronic disease (LBCD) [31] culminating in a final driver pathway, cardiovascular disease (e.g., atherosclerosis, heart failure, and atrial fibrillation)—a total of five cardiometabolic drivers. In these driver-based chronic disease models, obesity is ABCD stage 3, T2D is DBCD stage 3, and HTN is HBCD stage 3. The third dimension is the application of SDOH and cultural factors to each cell in the 4 × 5 (stage x driver) matrix to confer precision and optimal performance of clinical interventions aimed to prevent CMBCD progression. Validation efforts are underway to provide evidence corroborating the distinctness of each cell in the three-dimensional tCMBCD model based on specific stages, drivers, and populations [32–35]. Interventions are considered as preventive in nature, focusing on early stages (e.g., stages 1 and 2), and therefore centered on lifestyle change [27, 36]. Endpoints are mitigation of development and progression of tCMBCD stages, in terms of physiological, economic, and quality of life metrics.

In order to apply the tCMBCD model to forcibly displaced migrants, the cultural aspects of both the countries of origin and destination must be considered. The initial focus should be addressing the immediate needs of the most vulnerable forcibly displaced migrants by continuing the medical care and medications they had in their place of origin, which might have been disrupted by forced migration. The adoption of innovative prevention strategies must take into account the patient's health beliefs, such as understanding of T2D symptoms and complications, and how illness negatively impacts healthcare outcomes [37]. Compared to native population, refugees had a more limited ability to take care of themselves and self-monitor the progression of illness, relying more on HCPs. Additionally, the forcibly displaced population had poor dietary habits (e.g., continuation of improper past habits, acculturation of bad habits, and unfamiliarity with available foods) and suboptimal physical activity (e.g., lack of knowledge on how to exercise, lack of time, or cultural factors). Also, low socioeconomic status and level of education negatively impacted health literacy, which is an important factor in chronic disease outcomes [37].

A study evaluating 2,144 Venezuelan forcibly displaced migrants in Peru found that 57.2% reported non-utilization of healthcare services, more prevalent in younger migrants without chronic diseases, driven by lack of money or health insurance, self-medication, and lack of time [38]. In a study by Weller et al. [17] in 1474 migrants (57.1% undocumented, 18.2% asylum seekers) in the United Kingdom, asylum seeker status was associated with the highest risk (adjusted OR, 95% CI): 2.48, 1.48–4.14 of being denied healthcare in the National Health System, and being undocumented was associated with the highest risk of fearing arrest. A systematic review describing perceptions and attitudes of HCPs in managing care for migrants found that besides cultural and language barriers, other factors, such as limited institutional capacity and limited right to health care for illegal populations, made it difficult to meet healthcare needs [39]. In another review, migrants faced a lack of information regarding important aspects of the healthcare system, especially accessibility [40].

## The role of telehealth

“Telehealth” is defined as the use of electronic information and communication technologies to provide and support healthcare and services when distance separates the participants; “telemedicine” is a similar term restricted to the practice of medicine at distance. Telemedicine has evolved to include a wide variety of virtual medical services, such as online consultations, and the COVID-19 pandemic accelerated its adoption [41]. Recently, artificial intelligence-based telemedicine and the use of connected medical devices enable the collection of medical data in real-time and remote patient monitoring [42].

Telehealth is also an effective strategy to treat chronic metabolic diseases in various settings. Malacarne et al. [43] demonstrated the feasibility of a simplified telehealth application designed to decrease cardiovascular risk in remote areas of Italy. The project involved the use of trained nonmedical personnel to assist participants allowing the collection of CVD data and the transmission of the electrocardiograms from the remote site in Esino Lario to a central center in Milan to evaluate arrhythmias and cardiometabolic risk. Arrhythmias were found in 14% of subjects and high systolic (> 140 mmHg) and diastolic (> 90 mmHg) blood pressure was found in 43% and 31% of participants, respectively. One of the limitations of the aforementioned study is a sample of only 181 participants. This model was proposed as a strategy to provide cardiovascular care and screening programs in remote areas with low cost [43]. In Venezuela, since 1995, a telemedicine program (Maniapure Program) has increased the coverage of specialized healthcare in rural and indigenous areas [44]. The program has three levels of care: remote (basic clinic), virtual triage center (internist physicians or registered nurses receive the inquiry, examine the case, and provide adequate advice or refer to other specialists), and specialty level (e.g., for CMBCD drivers), each demonstrating effectiveness and significant cost reduction by avoiding unnecessary travel by the majority of consulting patients.

Despite studies limitations, telehealth strategies have demonstrated significant outcome benefits, which are comparable to those of traditional healthcare, but there are other benefits as well. A scoping review involving 4,960 participants analyzing the perceptions by patients of any ages in the USA, Canada, Australia, and the Pacific Islands in community centers, general practices, or outpatient services found that telehealth was considered feasible, satisfactory, and acceptable (particularly in a videoconference) [45]. The studied areas included mental health, diabetes, cancer, and other chronic conditions, demonstrating improvement in the care of remote and linguistically isolated populations. Other cited benefits of telehealth were convenience, lower cost, and adherence. Of the 17 studies included, two focused only on culturally and linguistically diverse patient groups, describing videoconferences as the preferred telehealth interventions for depression in Korean immigrants and for post-traumatic stress disorder in a rural multicultural group of Asian, Caucasian, and Pacific Islander participants [45]. Some of the limitations of the studies included in the aforementioned review that could impact the generalizability of results included small sample size, non-random selection, variation in the cultural values of groups included and limited number of ethnic groups included.

### Telehealth focus on diabetes and hypertension

T2D and HTN are two of the most prevalent chronic cardiometabolic diseases worldwide. A systematic review and meta-analysis evaluating the use of telemedicine (primarily monitoring and education) reported an absolute decline of hemoglobin A1c (A1C) by 0.35% in patients with T2D in telehealth programs compared to face-to-face care [46]. Other telemedicine benefits included a reduction in costs for the users and HCPs, as well as greater satisfaction; limitations however included heterogeneity in the analysis of the result. Moreover, a prospective cohort study of 104 no migrants patients in an urban setting receiving telemedicine for diabetes, HTN, and/or kidney disease treatment by a Federally Qualified Health Center found greater satisfaction and improved glycemic control with equivalent outcomes when compared with matched controls who received traditional care [47].

Dietary interventions can also impact T2D and HTN. Li et al. [48] using a simulation analysis previously validated for smoking cessation and nutrition promotion, projected the impact of expanding telehealth-delivered dietary interventions among older adults in Georgia, USA. They estimated that a telehealth-delivered dietary intervention could reduce the incidence of cardiometabolic risk, T2D, HTN, and high cholesterol [48]. As with all simulation models, one of the main limitations is the simplification of the real word. In the Get Healthy Service, a state-funded telephone-delivered coaching protocol in Australia, significant reductions in weight, body mass index, and A1C levels were found, compared to multidisciplinary care alone as part of a public obesity service [49]. Schrauben et al. [50] studied the implementation of a dietary app-supported tele counseling approach in 44 patients with T2D and chronic kidney disease stage 1 to 3A. The study used weekly motivational counseling performed by a registered dietitian for 8 weeks with follow-up evaluations at 6 and 12 months. The 12-month follow-up was achieved by 57% of participants reporting a decrease in sodium intake (by 638 mg/day from the baseline of 2,919 mg/day, *P* < 0.001), in systolic blood pressure (−5.7 mm Hg, 95%CI −10.5 to −1.0, *P* = 0.02), and in diastolic blood pressure (−4.1 mm Hg, 95%CI −7.2 to −1.1, *P* = 0.01), with an increase in Healthy Eating Index 2015 score. In contrast, the 24-h mean urine sodium and albumin excretion did not decline [50].

The COVID-19 pandemic represented an enormous challenge worldwide, including a decreased ability to control chronic diseases. Telehealth proved to be a feasible, sustainable, and effective tool to monitor T2D with social distancing imposed by the pandemic [51]. Some of the reported benefits of telehealth include an improvement in A1C and body weight during the lockdown, prevention of disruptions in filling medical prescriptions, and management of T2D complications such as retinopathy, depression, excess adiposity, and cost-effectiveness [52]. In Australia, a study evaluated the effect of government funded telemonitoring during the COVID-19 pandemic finding no significant differences in A1C levels when compared with face-to-face evaluations [53].

### Telehealth as a tool to change lifestyle

Interventions designed to improve health through lifestyle changes by increasing physical activity and loss of excess weight can be delivered by telehealth. A retrospective observational study of patients over 6 months of follow-up with medically managed obesity, divided into three cohorts (in-person visits only, in-person and video visits, or video visits only), found that telehealth was comparably effective to in-person visits for weight loss outcomes [54]. Moreover, the telehealth cohort had more visits than the in-person and hybrid cohorts, which could be related to greater engagement with HCPs by removing barriers to in-person visits. A randomized trial with a 12-month remotely delivered care consisting of 22 telephone calls, mailed material, and optional text messages to promote weight loss (diet and physical activity) in breast cancer survivors showed a significant decrease in weight, fat mass, metabolic syndrome, risk scores, waist circumference, and fasting plasma glucose, among others, when compared to usual care [55]. In another study, a multi-site, single-blinded, randomized controlled trial was performed to implement a lifestyle coaching telehealth program in acute, rehabilitation, and outpatient stroke units from four regional hospitals in British Columbia, Canada [56]. Although the program did not show significant improvement in participants' lifestyle behavior, it demonstrated an improvement in A1C levels and health-related quality of life among stroke survivors with mild stroke-related disabilities. It is important to notice that in all mentioned studies limitations included small sample size as well as a relatively short follow-up period.

### Cultural adaptations in telehealth

Effective telehealth incorporates cultural information about the target population and then adaptation when implementing strategic goals. For example, a study investigating the collective patterns of learning behaviors and preferences of Chinese patients with respect to diabetes education revealed a preference for prescriptive concrete instructions rather than flexible education, with professional healthcare treatment as a last resort [57]; this culturally determined attitude among Chinese migrants may be significantly different from prevailing attitudes in a country of destination. The use of mobile applications can illustrate how healthcare may be delivered in different ways to culturally different populations. A qualitative study performed in Swedish maternity care units found that some of the challenges in providing care to foreign-born pregnant women (Arabic and Somali) were a lack of resources within the clinical practice and a need for cultural awareness among HCPs [58]. This study reported that the use of a translated version of a previously evaluated Swedish app for maternity care was perceived as a helpful tool to support healthy lifestyle behaviors in foreign-born women. This result highlights the importance of cultural competence [59], that is, the recognition and inclusion of cultural dynamics to improve the interactions between patients and HCPs and, consequently, the effectiveness of healthcare delivery in diverse settings.

### Telehealth for migrants with type 2 diabetes and/or hypertension

**Fig. 1:**
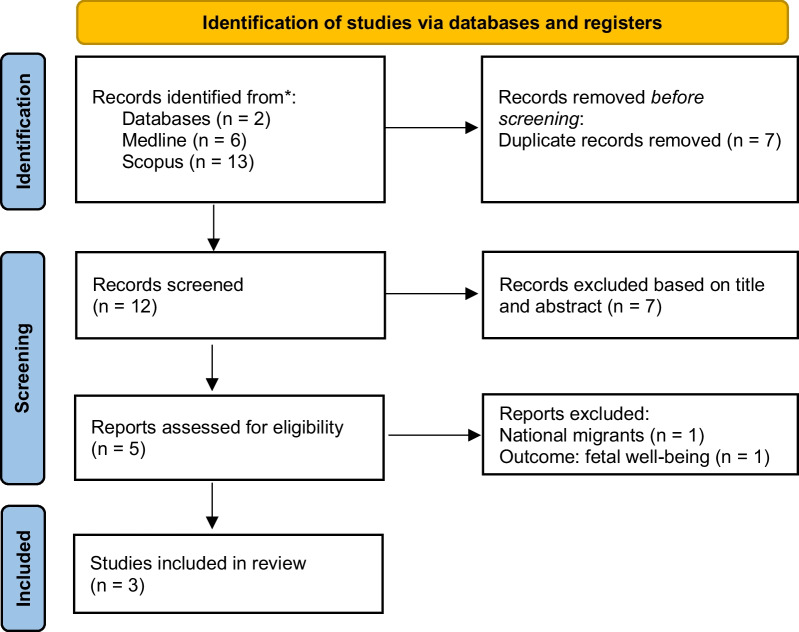
Selection of studies. PRISMA diagram

The effectiveness of using telehealth in difficult-to-reach forcibly displaced populations has been poorly studied. Our review included peer-reviewed articles of studies published in the databases MEDLINE and SCOPUS until February 2023, without restrictions of publication date or language (Fig. 1). The search was designed using key search terms “migrants”, “forcibly displaced populations”, “telemedicine”, “telehealth”, and “diabetes” or “hypertension”. The search identified 13 potentially relevant abstracts (three related to “hypertension”, and ten related to “diabetes”). Only studies including telemedicine interventions to screen or manage HTN or diabetes in migrant populations were included. Two reviewers (R.N.M and D.D.O) independently reviewed all titles and abstracts to determine if they met inclusion criteria. After abstract screening and retrieval of potentially relevant studies, six full-text publications were assessed for eligibility. Finally, three studies met the inclusion criteria [60–62] (Table 1).

**Table 1:** Telehealth intervention on diabetes and hypertension in migrants

Studies	Price et al. [60]	Foreman et al. [61]	Garner et al. [62]
Year	2011–2012	2022	2021
Objective	Determine rates of ownership of mobile devices and willingness to use mHealth strategies	To evaluate a bi-national consulate-based teleophthalmology screening service for diabetic retinopathy (DR) among Mexican migrants in the USA	Evaluate the use of a culturally relevant evidence-based application to improve health literacy in hypertension and type 2 diabetes among a migrant Indian subpopulation in Hong Kong
Target Population	Hispanic Migrant Farm Workers	Mexican Migrants in the United States	Asian Indian Migrants to Hong Kong
Location	Charleston County, South Carolina, USA	Mexican consulates in California	Hong Kong, China
Participants	80 Migrant Farm Workers, 70% male with a mean age of 29.8 years	508 adult visitors with self-reported diabetes at Mexican consulates in California	46 participants ≥ 18 years. Asian, Indian ethnicity born in India and migrated to Hong Kong
Methods	A questionnaire was designed to evaluate demographic information, essential hypertension status, and self-reported medication adherence. A series of 9 questions to assess attitudes toward remote monitoring for chronic disease management via mobile phone	Questionnaires and fundus photography. Photographs were graded for DR by retina fellows in Mexico via teleophthalmology	A mobile health application designed to improve hypertension and type 2 diabetes mellitus health literacy was tested using a mixed-methods design to determine its impact on improving health literacy in this subpopulation
Results	81% (65/80) owned cell phones capable of sending and receiving health-related messages. Most participants (65/80, 81%), were receptive to using mHealth technology. Relations between age and attitudes toward using mHealth were not statistically significant	97.6% of participants were aware that diabetes can cause vision loss. 24.4% had undergone an eye examination in the past year. Barriers to care were cost (53.9%) and insurance (45.6%). Most (85.4–91.1%) reported that Spanish-speaking providers and provision of screening in primary care would increase participation in screening. Any DR, vision-threatening DR, or proliferative DR were found in 30.2%, 9.9%, and 5.4% of participants, respectively. Nearly one-fifth (19.5%) received referrals	Quantitative findings indicated the mobile health application was effective in improving health literacy. Qualitative findings revealed participant perceptions about the application explored its informative nature, usability and likability of application components, and its ability to initiate intentionality for a healthier lifestyle among users

mHealthmobile health; DR—diabetic retinopathy.

In 2013, Price et al. [60] reported that more than 80% of Hispanic migrant farm workers in the USA had devices capable of sending and receiving telehealth information and were receptive to the idea of using this technology to receive health messages to improve monitoring of their diseases, treatment adherence, and medication changes. It has been shown that telehealth could improve HCPs cultural competency and awareness when caring for migrant populations. Foreman et al. [61] studied the use of teleophthalmology screening services for diabetic retinopathy in Mexican migrants in the USA using photographs that were graded by retina fellow’s doctors in Mexico. This study found that the key barriers to quality healthcare for Mexican migrants with diabetes included cost and by extension having insurance. The study also found that diabetic retinopathy existed in almost half of the sample with 19.5% receiving a referral for ophthalmological consultation; this affirms telehealth as a tool to increase the detection and treatment of diabetes complications. Garner et al. [62] studied the use of a culturally relevant evidence-based application to improve health literacy in T2D and HTN among a migrant Indian subpopulation in Hong Kong. After using the app, there was an improvement in T2D and HTN pretest and posttest mean scores. In the qualitative analysis, the participants reported the following positive aspects of the application: informativeness, usability, and likability. Moreover, the app users showed intentionality in making lifestyle changes to improve their health outcomes.

## Closing the practice gap: telehealth, lifestyle change, and cardiometabolic risk

Physical activity, healthy eating, and loss of excess adiposity are crucial lifestyle strategies that can reduce CVD risk, especially in vulnerable populations, such as the forcibly displaced migrant population. Although there are many reports about migrant population health, the use of telehealth to decrease the CMBCD burden in these groups has not yet been established.

Other than CMBCD and related conditions, telehealth has been used by migrants for other health services. A study was conducted to evaluate the use of a mobile application called Pregnancy and Newborn Diagnostic Assessment (PANDA) [63], a touchscreen application designed to provide antenatal care including the participation of migrant pregnant women. The system allowed the creation of electronic patient records and the identification of pregnancies as high-risk (10% of the sample). Additionally, they found that this mobile application could be useful to collect health information and provide comprehensive and high-quality antenatal care to migrant populations, which would facilitate the continuity of care for a population with frequent relocations. In 2020, the Non-Resident Nepali Association in collaboration with Danphe Care, who developed a virtual healthcare system based in Nepal, began providing virtual assistance to any person of Nepali origin around the globe through phone, text, or email [64]. Some of the most commonly consults received were for mental health and dermatological issues. Limitations included internet connectivity, technical illiteracy, and willingness to seek out these services. Riza et al. [65] studied the application of an electronic algorithm proposed to improve healthcare for migrants (especially refugees) by increasing health literacy and facilitating integration in countries of destination. This algorithm was applied to a sample of 82 migrants in reception and identification camps in Greece and found that assistance with mental health support, advice on vaccination, body weight control, and dental care was needed. The study also reported that more than half of the migrants had problems understanding medical information and did not know where to find healthcare for a specific concern. The project website provided migrants with access to sources and tools allowing them to find healthcare provision points and encouraging them to seek appropriate healthcare when necessary [65]. Culturally adapted telehealth services could be provided to migrant populations to improve healthcare and decrease the CMBCD burden.

## Conclusions

Forcibly displaced populations are migrants that left their countries of origin due to conflict, persecution, or environmental disasters, including refugees, asylum seekers, and undocumented persons. Migrants in general often face enormous health-care challenges determined by the baseline situation of their country of origin, travel difficulties, and health system barriers to accessing quality healthcare in the country of destination. Forcibly displaced migrants also have a higher prevalence of chronic conditions compared to the native population, including T2D and HTN. In several studies, telemedicine demonstrated outcomes comparable to those of traditional health care; importantly, there are limitations that need to be addressed future studies include generalizability of results to different populations and ethnic groups and small sample size. Overall, telehealth interventions could be implemented to improve the healthcare of difficult to reach populations such as forcibly displaced migrants. Telemedicine can potentially decrease the burden of chronic conditions such as HTN and T2D of these vulnerable populations by improving lifestyle as part of the tCMBDC model, with the additional benefit of reducing healthcare associated costs. More prospective studies implementing telemedicine strategies and affordable telemedicine services to treat cardiometabolic disease burden in migrant populations are needed.

## Data Availability

Not applicable.

## Abbreviations

ABCD: Adiposity-Based Chronic Disease
CVD: Cardiovascular Disease
CI: Confidence interval
DR: Diabetic Retinopathy
DBCD: Dysglycemia-Based Chronic Disease
HCPs: Healthcare Professionals
A1C: Hemoglobin A1c
HTN: Hypertension
HBCD: Hypertension-Based Chronic Disease
LBCD: Lipid-Based Chronic Disease
mHealth: Mobile health
OR: Odds ratio
SDOH: Social Determinants of Health
tCMBCD: Transcultural Cardiometabolic-Based Chronic Disease
T2D: Type 2 diabetes
